# Supplementary material for: PIEZO1 regulates leader cell formation and cellular coordination during collective keratinocyte migration
Source: PLoS Comput Biol. 2024 Apr 5;20(4):e1011855. doi: 10.1371/journal.pcbi.1011855 (PMC11023636; doi:10.1371/journal.pcbi.1011855)
Supplement: S1 Text — (1) Boundary conditions of governing equation. (2) Initial condition of governing equation. (3) Positive definite diffusivity. (4) Retraction is modeled by advection. (5) Function smoothing. (6) Model dimensionalization. (7) Alternative model: a fully continuum approach. (8) Robustness testing for model calibration. (9) Heterogeneous cell collective migration model. (PDF) [file pcbi.1011855.s001.pdf]

S1 Text

Section 1. Boundary conditions of governing equation

On the Dirichlet boundaries $y = 0$ and $y = 1$ (Eq. 17), the cell density is determined by functions $g_1(x, t)$ and $g_2(x, t)$ which are continuous on $[0, 1] \times [0, +\infty)$. Since both of these are randomly generated from the same approach, without loss of generality, let's say $g(x, t)$. Covering $[0, 1] \times [0, +\infty)$ with a grid, taking mesh sizes h_x and h_t and labeling grid nodes $(x, t) = (x_i, t_j) = (ih_x, jh_t)$ by indices (i, j), the function values at grid points $g_{i,j} = g(x_i, t_j)$ are taken to follow a normal distribution

$$g_{i,j} \overset{iid}{\sim} \mathcal{N}(\mu_0, \sigma_0^2) \tag{27}$$

with mean μ_0 and standard deviation σ_0 ($\mu_0 = 0.6$ and $\sigma_0 = 0.3$ were adapted in the simulation). This models the variability of the influx of cells from the monolayer moving into the wound region. Thereafter, the function $g(x, t)$ is given by an interpolation on $g_{i,j}$. Specifically, the boundary conditions on $y = 0$ and $y = 1$ are classical Dirichlet boundary conditions with a constant influx μ_0 if σ_0 is set to be 0.

Section 2. Initial condition of governing equation

Assume $u(x, y, t)$ is a function defined on $[0, 1] \times [0, 1] \times [0, +\infty)$ and satisfies the following diffusion equation

$$\frac{\partial u}{\partial t} = \nabla \cdot (\boldsymbol{D} \nabla u) \tag{28}$$

with the same diffusivity $\boldsymbol{D}$ as in Eq. 5 and the same boundary conditions as in Eq. 17:

$$\begin{gathered} u(x, 0, t) = g_0(x, t), \quad u(x, 1, t) = g_1(x, t), \\ \frac{\partial u(0, y, t)}{\partial x} = \frac{\partial u(1, y, t)}{\partial x} = 0, \end{gathered} \tag{29}$$

while the initial condition is globally zero:

$$u(x, y, 0) \equiv 0. \tag{30}$$

With this setting, the wound region ($u = 0$) is narrowing down from the whole square domain $[0, 1] \times [0, 1]$ to a heterogeneous horizontal banded region in the middle of the domain, before finally shrinking to zero area and disappearing at $t = t_{end}$. At a certain time point $t = t_0 \in (0, t_{end})$ during this process,we set $\rho(x, y, 0) = u(x, y, t_0)$ as the initial condition of our governing equation Eq. 4.

In other words, this initial condition is generated by the governing equation (Eq. 4) but without retraction, starting from zero initial values and diffusing cells without any retraction for a period of time, until retractions were introduced. At the moment right before the first retraction, cell densities across the domain $[0, 1] \times [0, 1]$ are the initial values for the governing equation. This enables us to start with a variable, and more physiological, initial condition compared to taking a constant values at the wound edge.

Section 3. Positive definite diffusivity

The matrix $d \cdot (w_I \boldsymbol{I} + w_A \boldsymbol{A})$ is diagonal and has a positive spectrum. Therefore, the diffusivity $\boldsymbol{D} = d \cdot (w_I \boldsymbol{I} + w_A \boldsymbol{A}) \cdot \hat{D}_\alpha(\rho)$ of the governing equation (Eq. 4) is positive definite if and only if the scalar diffusion coefficient $\hat{D}_\alpha(\rho) > 0$, which depends on the value of adhesion coefficient α. By inspecting this 5-th degree polynomial, we see that $\hat{D}_\alpha(\rho) > 0$ unconditionally holds for all levels of cell density $\rho \in (0, 1)$ as long as

$$\alpha < \hat{\alpha} := \frac{1}{17 - 4\sqrt{15}} \approx \frac{2}{3} \tag{31}$$

with a critical value $\hat{\alpha}$. When $\alpha \geq \hat{\alpha}$, there exists an interval

$$I_\alpha = \left(\frac{1 + 7\alpha - \sqrt{1 - 34\alpha + 49\alpha^2}}{12\alpha}, \frac{1 + 7\alpha + \sqrt{1 - 34\alpha + 49\alpha^2}}{12\alpha} \right) \tag{32}$$

such that $\hat{D}_\alpha(\rho) < 0$ if and only if $\rho \in I_\alpha$. That is, the diffusivity is negative definite when cell density $\rho \in I_\alpha$, which results in the ill-posedness of the initial value PDE problem. As $\alpha \to 1$, the interval I_α expands from a single point $17 + 4\sqrt{15} \approx 1.5$ to $I_1 = (1/3,\ 1)$.

Section 4. Retraction is modeled by advection

Performing a Taylor expansion on the cell density ρ, centered at $\boldsymbol{x} = \boldsymbol{x}_{i,j}$, in the discrete master equation (Eq. 1) without specifying $b^{\rightarrow}_{i,j}$, we have

$$\frac{\partial \rho}{\partial t} = \nabla \cdot (\boldsymbol{D} \nabla \rho + h \cdot (1 - \rho)(1 - \alpha\rho)^3 \cdot (b^{\leftarrow} - b^{\rightarrow}, b^{\uparrow} - b^{\downarrow})^T) + \mathcal{O}(h^2) \tag{33}$$

where $\boldsymbol{D}$ is the same diffusivity as in Eq. 5. By taking $h \to 0$, the continuum limit would be a simple diffusion equation $\partial\rho/\partial t = \nabla \cdot (\boldsymbol{D}\nabla\rho)$ without an advection term, unless both $\Delta b^{\leftrightarrow} = b^{\leftarrow} - b^{\rightarrow}$ and $\Delta b^{\updownarrow} = b^{\uparrow} - b^{\downarrow}$ are $\mathcal{O}(1/h)$, the advection scaling. Therefore, we define $b^{\rightarrow}_{i,j} := r^{\rightarrow}_{i,j}/h$ with $r^{\rightarrow}_{i,j} \in \mathcal{O}(1)$. By taking $h \to 0$ under this setting, Eq. 33 turns into our continuum limit (Eq. 4), where the retraction is modeled by advection.

Section 5. Function smoothing

To localize the retraction region (Eq. 10), we smooth the Heaviside function $H(\gamma - \rho)$ (Eq. 11) using a hyperbolic tangent function

$$\tilde{H}(\gamma - \rho) = \frac{1}{2} \cdot (1 + \tanh(k \cdot (\gamma - \rho))), \tag{34}$$

where k is the steepness level at transition point $\rho = \gamma$ ($k = 10$ was adapted in the simulations). On the other hand, the indicator function $\mathbb{1}_{\Omega_i}(\boldsymbol{x})$ is smoothed using a 2D generalized bell-shaped function:

$$\tilde{\mathbb{1}}_{\Omega_i}(\boldsymbol{x}) = \frac{1}{1 + (\frac{dist(\boldsymbol{x},\Omega_i)}{k_1})^{2k_2}}, \tag{35}$$

where k_1 and k_2 are parameters determining the width and steepness of the transition region in the smoothing process. The distance between a point $\boldsymbol{x}$ and a set Ω_i in 2D Euclidean space is induced by a natural 2-norm $\|\cdot\|$:

$$dist(\boldsymbol{x}, \Omega_i) := \inf_{\boldsymbol{y} \in \Omega_i} \|\boldsymbol{x} - \boldsymbol{y}\|. \tag{36}$$

Since the region Ω_i is banded, the indicator function $\mathbb{1}_{\Omega_i}(\boldsymbol{x})$ is equivalent to its 1D form $\mathbb{1}_{[c_i - \omega_r/2, c_i + \omega_r/2]}(x)$ (Eq. 12). Therefore, the 2D generalized bell-shaped function $\tilde{\mathbb{1}}_{\Omega_i}(\boldsymbol{x})$ (Eq. 35) can be simplified into a 1D version:

$$\tilde{\mathbb{1}}_{[c_i - \omega_r/2, c_i + \omega_r/2]}(x) = \frac{1}{1 + \left| \frac{x - c_i}{k_1 \omega_r} \right|^{2k_2}}. \tag{37}$$

By adjusting the center and the width of the characteristic interval, the generalized bell-shaped function given above can be applied to smooth the indicator function in time $\mathbb{1}_{[\tau_i,\tau_i+\mathcal{T}_i)}(t)$ as the following:

$$\tilde{\mathbb{1}}_{[\tau_i,\tau_i+\mathcal{T}_i)}(t) = \frac{1}{1+\left|\frac{t-\tau_i-\frac{1}{2}\mathcal{T}_i}{k_1\mathcal{T}_i}\right|^{2k_2}}. \tag{38}$$

Since the spatial regions of retractions decay away before the next retraction event occurs, shifts in the retraction region $c_i \sim \mathcal{U}(0,1)$ do not introduce discontinuities. Note that the selection of width and steepness parameters (k_1 and k_2) for smoothing indicator functions are different for the spatial and temporal localizations of retraction (S4 Fig).

Section 6. Model dimensionalization

Recall that Eq. 1 is our non-dimensional master equation with the transitional probability Eq. 2. In order to relate the dimensions in the model to the experiments, we take the dimensional variables to be (1) $\hat{\rho}_{i,j} := \rho_{\max}\cdot\rho_{i,j}$ ($\rho_{\max}$ is the maximal cell density), (2) $\hat{h} := l\cdot h$ (l is the characteristic length) and (3) $\hat{t} := \lambda^{-1}\cdot t$ (λ^{-1} is the characteristic time). Hence, the dimensional transitional probability becomes

$$\hat{T}_{i,j}^{\rightarrow} = (1-\tfrac{\hat{\rho}_{i+1,j}}{\rho_{max}})(1-\alpha\tfrac{\hat{\rho}_{i-1,j}}{\rho_{max}})(1-\alpha\tfrac{\hat{\rho}_{i,j+1}}{\rho_{max}})(1-\alpha\tfrac{\hat{\rho}_{i,j-1}}{\rho_{max}})(d^{\leftrightarrow}\cdot\tfrac{\hat{\rho}_{i,j}}{\rho_{max}}\cdot\tfrac{l^2}{\hat{h}^2}+r_{i,j}^{\rightarrow}\cdot\tfrac{l}{\hat{h}}), \tag{39}$$

which can be taken into the master equation (Eq. 1) with the dimensional time derivative

$$\frac{\partial\rho}{\partial t} = \frac{\partial(\frac{\hat{\rho}}{\rho_{max}})}{\lambda\hat{t}} = \frac{1}{\lambda\rho_{max}}\cdot\frac{\partial\hat{\rho}}{\partial\hat{t}} \tag{40}$$

and obtain the continuum limit by taking $\hat{h}\to 0$:

$$\frac{\partial\hat{\rho}}{\partial\hat{t}} = \nabla\cdot(\tilde{\boldsymbol{D}}\nabla\hat{\rho}) + \nabla\cdot(\tilde{\boldsymbol{R}}\hat{\rho}). \tag{41}$$

Here, $\tilde{\boldsymbol{D}}$ is the dimensional diffusivity (diffusion coefficient) given by

$$\tilde{\boldsymbol{D}} = \frac{\lambda l^2}{\rho_{max}^5}\cdot d\cdot(w_I\boldsymbol{I}+w_A\boldsymbol{A})\cdot\tilde{D}_\alpha(\hat{\rho}) \tag{42}$$

with

$$\begin{aligned}\tilde{D}_\alpha(\hat{\rho}) =& 2\rho_{max}^4\hat{\rho} - (1+11\alpha)\rho_{max}^3\hat{\rho}^2 + (8\alpha+16\alpha^2)\rho_{max}^2\hat{\rho}^3 \\ &- (13\alpha^2+7\alpha^3)\rho_{max}\hat{\rho}^4 + 6\alpha^3\hat{\rho}^5,\end{aligned} \tag{43}$$

and $\boldsymbol{I} = \boldsymbol{I}_2$, $\boldsymbol{A} = diag(0,1)$ are defined as before in Eq. 5. On the other hand, $\tilde{\boldsymbol{R}}$ is the dimensionalized retraction (advection velocity) given by

$$\tilde{\boldsymbol{R}} = \frac{\lambda l}{\rho_{max}^4}\cdot(\rho_{max}-\hat{\rho})(\rho_{max}-\alpha\hat{\rho})^3\cdot(\Delta r^{\leftrightarrow},\Delta r^{\updownarrow})^T \in \mathbb{R}^2 \tag{44}$$

where $\Delta r^{\leftrightarrow}$ and $\Delta r^{\updownarrow}$ are defined as in Eq. 10.

In order to connect the model with the experiments and to calculate the effective cell diffusion coefficient as well as the advection velocity, we need to know 3 parameters: λ, l and $\rho_{\max}$. We notice that $\lambda l^2 = v\cdot l$, where $v := \lambda l$ is actually the characteristic velocity (length over time). Hence, if we have a measurement of the characteristic velocity and the length scale, we can determine the characteristic time by $\lambda = v/l$. In

conclusion, we can connect our theory and numerical parameters with the biological experiments in the following way:

- Maximal cell density $\rho_{\max}$ and dimensional cell density $\hat{\rho}$: here $\hat{\rho}$ is interpreted as a number density, i.e., $\hat{\rho}\ dxdy$ is the number of individuals with the position in the phase area $dxdy$ centered at (x, y). We can quantify this from the experimental results: put down a grid, count the number of cells in each single square and get a spatial representation of the cell density. In the monolayer region away from the front edge, we expect the cell density should be nearly uniform spatially and temporally, and that value could be used for $\rho_{\max}$.
- Characteristic length l: we define the characteristic length scale to be the distance from the wound edge to the region where the cells reach the maximal density in the monolayer. In our numerical tests, we did not simulate the whole experimental domain, instead, our simulation focused on the region of transition, that is, the region in which the cell density transits from the front to the maximum. Hence, our computational domain is a small region around the wound edge ($\sim$10 cell lengths).
- Characteristic velocity v: the velocity of the moving front can be used for this, by averaging the front advancing speed measured by cell shape analysis.
- Characteristic time λ^{-1}: since we already have the way to determine the characteristic length l and velocity v, the characteristic time can be derived directly by $\lambda^{-1} = l/v$.

With the measurements mentioned above, we are able to calculate $\rho_{\max}$, l, v, λ and hence the diffusion coefficient and retraction velocity. At this point, we do not have a direct measurement for the adhesion coefficient α. A direct measurement for the cell-cell adhesion is being considered in our future work.

Section 7. Alternative model: a fully continuum approach

Recall that our model was initially formulated at the discrete level and subsequently upscaled into a continuous PDE. Here, we test a phenomenological continuum model in which cell-cell adhesion is postulated at the continuum level rather than being obtained by upscaling.

In this new model, the governing equation is still a diffusion-advection equation in the same form as Eq. 4, but the hindering effect of cell-cell adhesion on collective migration is modeled by reducing the overall diffusion coefficient as adhesion increases, which is consistent with the approach used by Amereh *et al.* in [1]. While still accounting for coordinated directionality, volume filling effects and the advancing front connecting the wound and the monolayer, the diffusion coefficient in this new continuum model can be specifically expressed as

$$\boldsymbol{D} = d \cdot (w_I \boldsymbol{I} + w_A \boldsymbol{A}) \cdot \hat{D}(\rho), \tag{45}$$

where the overall structure mirrors the diffusion coefficient in our original model (Eq. 5). However, the scalar diffusion coefficient, denoted as $\hat{D}(\rho)$, becomes a quadratic polynomial of cell density without depending on any additional parameters:

$$\hat{D}(\rho) = 2\rho - \rho^2. \tag{46}$$

Compared with the original model, d in Eq. 45 now contains the combined effects of cell motility and cell-cell adhesion.

Analogously, we assume that the advection velocity would now model the combined effects of retraction strength and cell-cell adhesion. In particular, the advection term in Eq. 4 now becomes

$$\boldsymbol{R} = (1-\rho) \cdot (\Delta r^{\leftrightarrow}, \Delta r^{\updownarrow})^T \tag{47}$$

where $\Delta r^{\leftrightarrow}$ and $\Delta r^{\updownarrow}$ are defined in the same way as in Eq. 10 but incorporating the combined effects of retraction strength and cell-cell adhesion.

To compare the model predictions with our original model, we replicated all the previous simulations related to *Piezo1*-cKO, *Piezo1*-GoF and Yoda1 using this new phenomenological model. Similar to the calibration process for the original model, we adjusted model parameters based on experimental data (S5 Fig). This involved varying model parameters from a wild type (e.g., $\text{Control}_{\text{GoF}}$) to a PIEZO1 phenotype (e.g., *Piezo1*-GoF), and measuring changes of wound healing metrics (wound closure and edge length) from repeated simulations. In line with our previous findings, we observed that the simulation results from the $\text{Control}_{\text{GoF}}$ to *Piezo1*-GoF case are only able to replicate experimental observations if coordinated directionality is reduced. That is, by reducing the parameter of coordinated directionality, we recapitulated the experimental phenotype of both a shorter edge length and slower wound closure in simulated *Piezo1*-GoF monolayers (S14A Fig). Importantly, we noted that changes to the diffusion coefficient, according to changes in cell-cell adhesion, alone failed to replicate all experimental results (S15 Fig), consistent with the results obtained using the upscaled model of adhesion. This underscores the primary role of coordinated directionality in PIEZO1's impact on reepithelialization and reaffirms our main conclusion that PIEZO1 activity hinders coordinated directionality. Because the dependence of the diffusion coefficient and the retraction strength on cell-cell adhesion could be quite different quantitatively, as suggested by our upscaled model (see Eq. 8 and Eq. 9), for simplicity, here we focused only on the changes in d and not on $\Delta r^{\leftrightarrow}$ and $\Delta r^{\updownarrow}$. However, we varied the retraction strengths and durations in the context of the original upscaled model (see Section 8 in S1 Text for details) and reached the same conclusion.

Section 8. Robustness testing for model calibration

The experimental data used for model calibration can be categorized into two main components: cell motility and retraction processes (including retraction duration, inter-retraction duration, and retraction strength). In the process of model calibration, we utilized experimental data at the single-cell level from Table 2 and S5 Fig. To test whether our conclusions depend on the quantitative single cell data, we varied the magnitudes of the motility and retraction processes.

For cell motility, we used experimental data from the monolayers (S9 Fig), which shows that cell motility within the monolayer increased in *Piezo1*-cKO and decreased in *Piezo1*-GoF and Yoda1-treatment compared to their respective experimental controls. Calibrating our original model using motility measured from monolayers, together with the original magnitudes of the retraction processes, and maintaining cell-cell adhesion and coordinated directionality as observed in $\text{Control}_{\text{GoF}}$, we found that while we could replicate simulated monolayers of *Piezo1*-GoF keratinocytes exhibiting slower wound closure compared to simulated $\text{Control}_{\text{GoF}}$ monolayers, but we failed to observe the decrease in simulated monolayer edge length seen in experiments (S16A Fig). However, by reducing the parameter of coordinated directionality, we recapitulated the experimental phenotype of both a shorter edge length and slower wound closure in simulated *Piezo1*-GoF monolayers (S16A Fig). On the other hand, our model simulations, calibrated using the monolayer cell motility dataset along with original

retraction processes for both *Piezo1*-cKO and Yoda1-treated keratinocytes, reproduced the observed experimental trends. However, with adjustments to coordinated directionality, we observed a more pronounced effect (S16B and S16C Fig). Importantly, we noted that changes to cell-cell adhesion parameters alone failed to replicate all experimental results, underscoring the primary role of coordinated directionality in PIEZO1's impact on reepithelialization (S17 Fig).

For retraction processes (retraction duration, inter-retraction duration and retraction strength), our previous work [2] indicated qualitative consistency in between single cell data and monolayer experiments. For example, in both single cell and monolayer experiments, retractions in the presence of Yoda1 consistently exhibit shorter durations and larger magnitude retractions compared to DMSO, albeit with variations in the degree of change. Building upon this qualitative observation from experiments, we conducted additional simulations. In these simulations, we recalibrated our original model based on general qualitative trends rather than specific quantitative values for retraction. For instance, Yoda1-treated cells exhibit significantly stronger retraction, with a strength approximately three times (around 2.87) that of its control DMSO-treated cells (S5 Fig). Instead of adhering strictly to this specific ratio of 2.87, we performed simulations using two additional ratios, one larger and and one smaller, while maintaining the qualitative trend in which the retraction strength in Yoda1-treated cells is greater than in DMSO-treated cells. This approach was also applied to the calibration of the retraction duration and the inter-retraction duration, with adjustments made in various ratios rather than relying on specific quantitative values derived from experimental statistics (S5 Fig). Again, two additional values of the durations were used.

Applying this recalibration to simulated monolayers of *Piezo1*-GoF keratinocytes, we observed slower wound closure compared to $\mathrm{Control_{GoF}}$ monolayers; however, the expected decrease in simulated monolayer edge length was not observed (S18 Fig). Consistent with our previous findings, simulations from the $\mathrm{Control_{GoF}}$ to *Piezo1*-GoF case could replicate experimental observations only when coordinated directionality was reduced. Specifically, by lowering the parameter of coordinated directionality, we recapitulated the experimental phenotype of both a shorter edge length and slower wound closure in simulated *Piezo1*-GoF monolayers, while changes to cell-cell adhesion parameters failed to replicate all experimental results (S18 Fig). The results from this recalibrated model reaffirm the role of coordinated directionality, leading to the same fundamental conclusion that PIEZO1 activity hinders coordinated directionality.

Section 9. Heterogeneous cell collective migration model

We expanded the original model to explore the influence of PIEZO1 activity in mixed populations. This model considers the migration of two distinct cell types, each governed by its own set of equations and parameters, while interacting through cell-cell adhesion and volume-filling effects. Specifically, the cell densities are denoted as u and v, and hence there are three types of cell-cell adhesions: (1) between u cells (α_{uu}), (2) between v cells (α_{vv}), and (3) interaction between u cells and v cells (α_{uv}). We assume that the interaction adhesion α_{uv} hinders collective cell migration in the same way as α_{uu} and α_{vv}. Regarding volume-filling effects, the migration of either u cells or v cells is impaired by the total density of cells $(u + v)$ in the front. The spatial position of wound edges are determined by the interface connecting the total cell $u + v$ and the cell-free region. However, retractions near wound edges are applied individually for u cells and v cells, following their respective retraction parameters, including the duration and strength of retraction, as well as the inter-retraction duration.

Following the same framework as our original homogeneous cell model, the heterogeneous collective cell migration occurs within a square domain, which is defined

by two opposite sides with zero-flux Neumann boundaries and two opposite sides with randomized Dirichlet boundaries. Source cells migrating into the domain from randomized Dirichlet boundaries exhibit stochastic influx and stochastic proportions of cells, with the mean of the v cell proportion among source cells designated as p_v. The initial distribution of total cells $u + v$ aligns with our original model (see Section 2 in S1 Text), where cells are evenly mixed with a p_v percentage of v cells and consequently, a $1 - p_v$ percentage of u cells.

To simulate monolayers with a mixture of $\text{Control}_{\text{cKO}}$ and *Piezo1*-cKO cells, we used parameters for the $\text{Control}_{\text{cKO}}$ and *Piezo1*-cKO cells from the homogeneous monolayers we simulated previously. This ensures that u cells exhibit migration behavior akin to $\text{Control}_{\text{cKO}}$, while v cells mirror the characteristics of *Piezo1*-cKO. Throughout this mixed collective migration process, we varied the proportion of *Piezo1*-cKO cells in initial and source cells (p_v), and we observed that cases with a higher *Piezo1*-cKO percentage displayed faster wound closure (S19A Fig), which is consistent with the faster wound closure observed in homogeneous *Piezo1*-cKO monolayers. Additionally, *Piezo1*-cKO cells tended to advance to the front and aggregate around the wound edge (S19A Fig).

To quantify this effect, we measured the percentage of *Piezo1*-cKO cells among all wound edge cells at every time step during the wound closure process, and subsequently calculated the average. The term "wound edge cells" pertains to cells situated near the wound edge, where the total density satisfies the condition $0 < u + v < \gamma_{\text{edge}}$. To determine the threshold γ_{edge} for identifying wound edge cells, we conducted simulations by mixing $\text{Control}_{\text{cKO}}$ and $\text{Control}_{\text{cKO}}$ rather than mixing $\text{Control}_{\text{cKO}}$ and *Piezo1*-cKO. That is, both u and v cells have the identical phenotype and parameters drawn from the $\text{Control}_{\text{cKO}}$ experimental data. In this case, for any fraction of v in the entire monolayer, we expect to observe the same fraction of v cells in wound edge cells where $u + v < \gamma_{\text{edge}}$. The minimal γ_{edge} that satisfies this condition is the threshold we sought, and it was determined to be 0.2. The results revealed that *Piezo1*-cKO cells are over-represented at the wound edge (S19B Fig).

Analogously, we investigate the mixing of $\text{Control}_{\text{GoF}}$ and *Piezo1*-GoF. Guided by the findings from our original model, in addition to adjusting retraction-related parameters and cell motility based on experimental data (S5 Fig), we also decrease the parameter of coordinated directionality in *Piezo1*-GoF (v cells) compared to $\text{Control}_{\text{GoF}}$ (u cells). By varying p_v, representing the proportion of *Piezo1*-GoF cells in initial and source cells, we observed that scenarios with a higher *Piezo1*-GoF percentage exhibited slower wound closure (S19C Fig), which is consistent with the slower wound closure observed in homogeneous *Piezo1*-GoF monolayers. Quantitative analysis revealed that, in contrast to the *Piezo1*-cKO case, *Piezo1*-GoF cells are underrepresented at the leading edge of the monolayer (S19C and S19D Fig).

Further insights into this heterogeneous cell migration model, a comprehensive exploration of the intricacies and findings derived from this study, will be provided in future work.

References

1. Amereh M, Edwards R, Akbari M, Nadler B. In-Silico Modeling of Tumor Spheroid Formation and Growth. Micromachines. 2021;12(7):749.

2. Holt JR, Zeng WZ, Evans EL, Woo SH, Ma S, Abuwarda H, et al. Spatiotemporal dynamics of PIEZO1 localization controls keratinocyte migration during wound healing. Elife. 2021;10.
