# Supplementary figures and images for: PIEZO1 regulates leader cell formation and cellular coordination during collective keratinocyte migration
Source: PLoS Comput Biol. 2024 Apr 5;20(4):e1011855. doi: 10.1371/journal.pcbi.1011855 (PMC11023636; doi:10.1371/journal.pcbi.1011855)

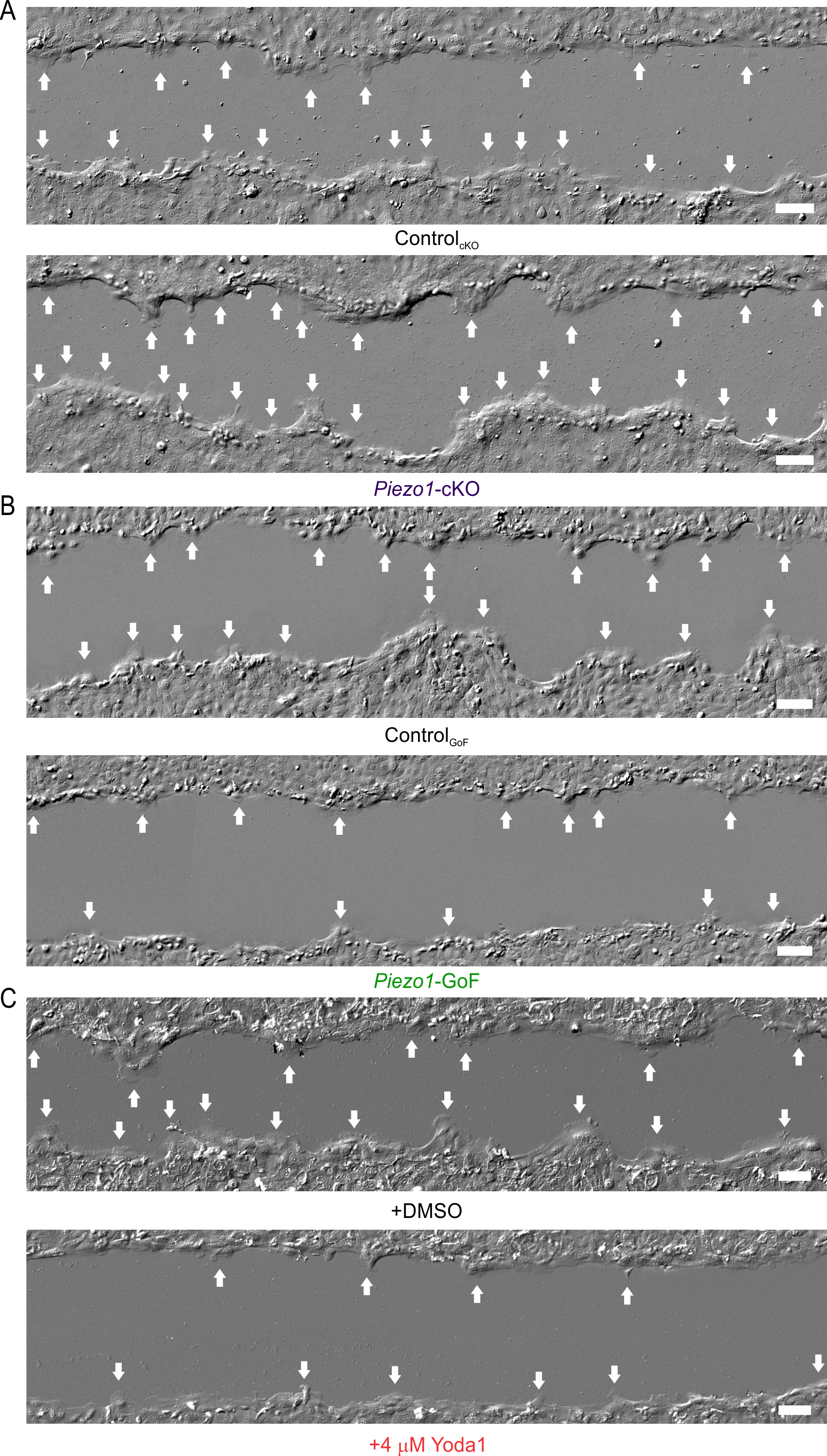

Supplement: S1 Fig — Representative DIC images of wounds generated in (A; top) ControlcKO, (A;bottom) Piezo1-cKO, (B; top) ControlGoF, (B; bottom) Piezo1-GoF, (C; top) DMSO-treated and (C; bottom) 4 μM Yoda1-treated monolayers. White arrows indicate leader cell protrusions. Representative images were taken at the same time point as the respective control field of view. Scale bar = 100 μm. Related to Fig 1. (TIFF) [file pcbi.1011855.s002.tiff]

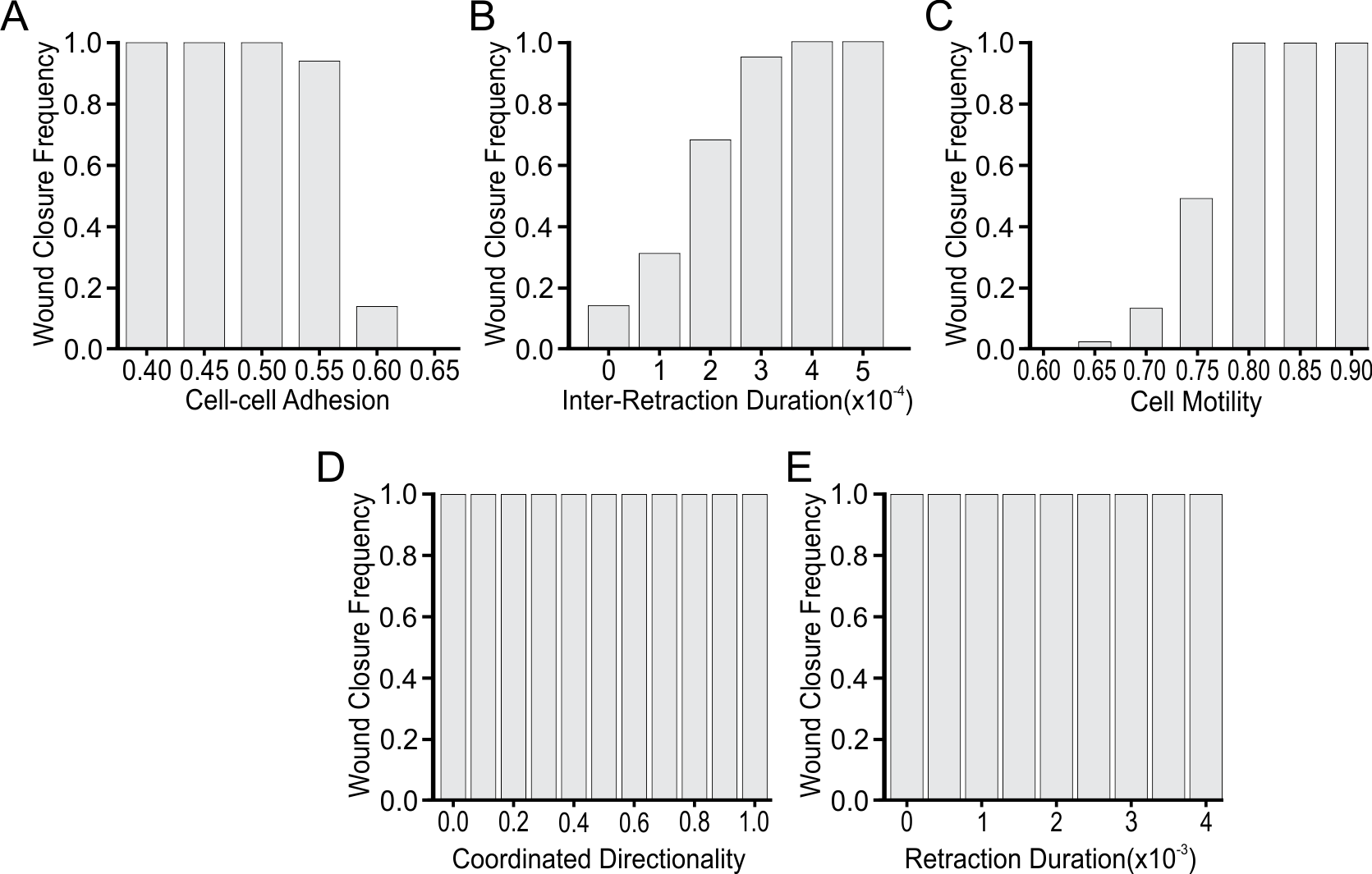

Supplement: S2 Fig — (A) The percentage of wound closure cases under different levels of cell-cell adhesion. (B, C, D, E) Similar to (A) but for inter-retraction duration, cell motility, coordinated directionality and retraction duration respectively. Related to Fig 2F. (TIFF) [file pcbi.1011855.s003.tiff]

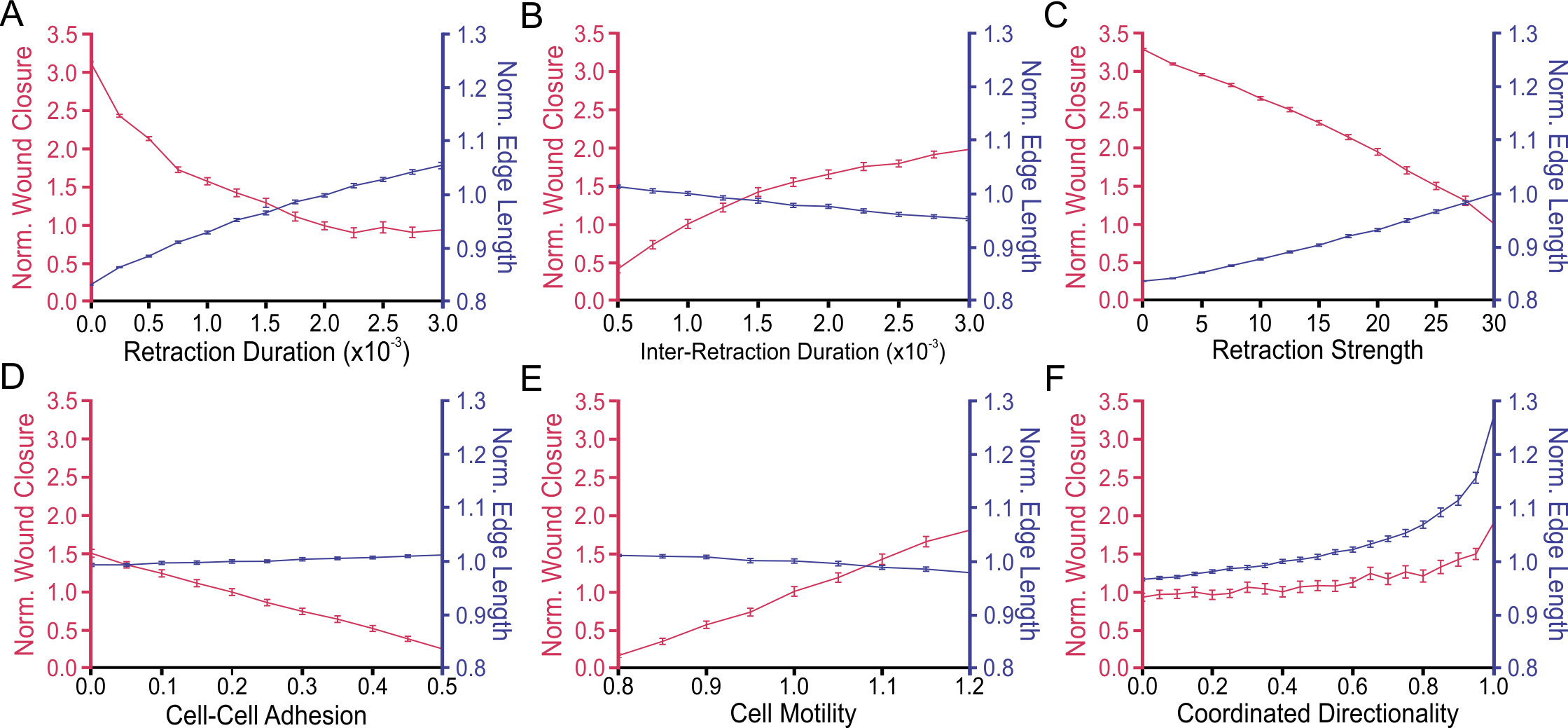

Supplement: S3 Fig — (A) The mean of 100 simulation results showing the effect of retraction duration on normalized wound closure (red; left axes) and edge length (blue; right axes). Error bars depict the standard error of mean. (B-F) Similar to (A) but for inter-retraction duration, retraction strength, cell-cell adhesion, cell motility and coordinated directionality, respectively. The data in C and F are also shown in Fig 2G and 2H but are reproduced here for ease of comparison. (TIFF) [file pcbi.1011855.s004.tiff]

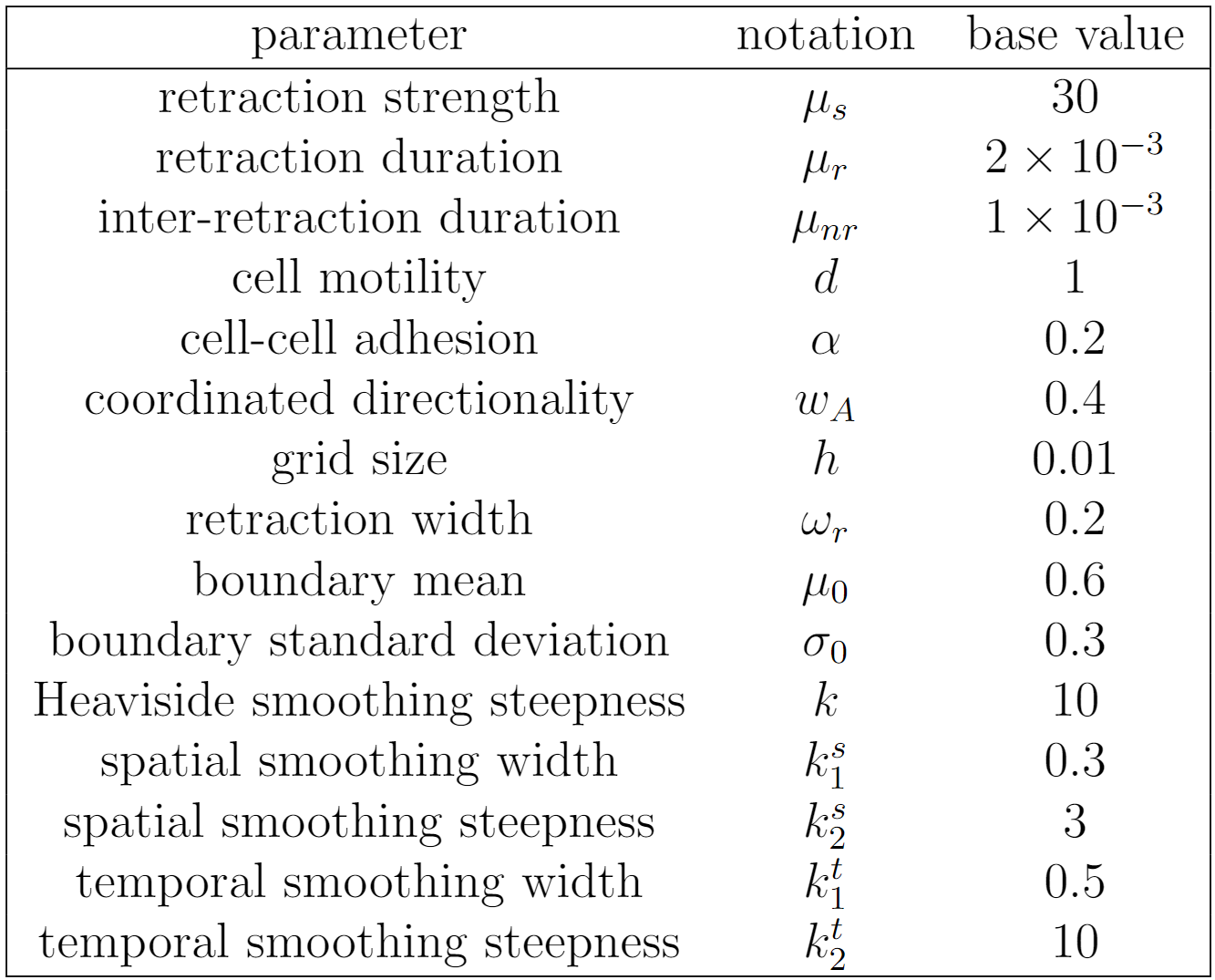

Supplement: S4 Fig — (TIFF) [file pcbi.1011855.s005.tiff]

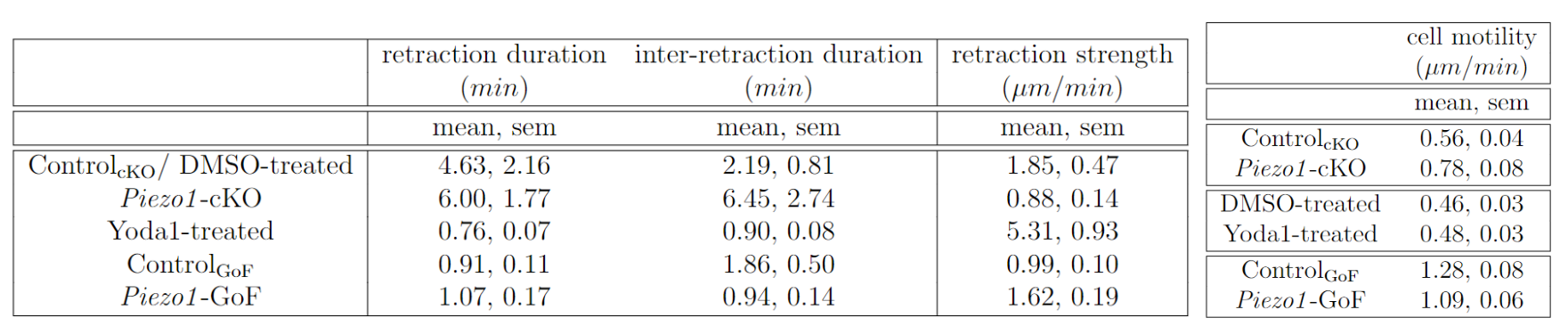

Supplement: S5 Fig — Mean and standard error of mean (sem) of single cell migration dataset (retraction duration, inter-retraction duration, retraction strength and cell motility) under different experimental conditions. Retraction duration data was derived by kymograph measurements, retraction strength derived from cell shape analysis and cell motility data from tracking cells during single cell migration assays [22]. (TIFF) [file pcbi.1011855.s006.tiff]

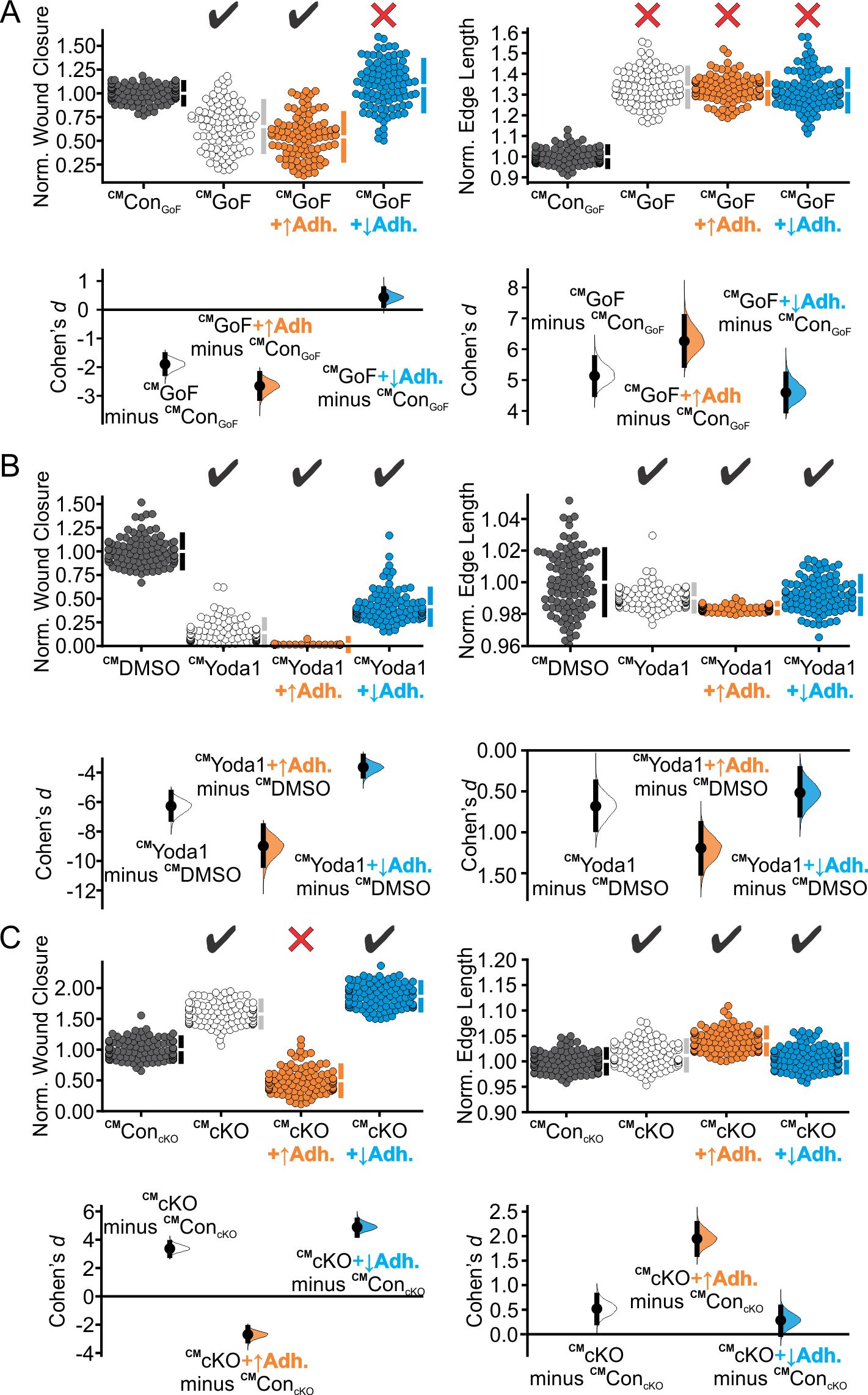

Supplement: S7 Fig — (A) Cumming plots showing simulation results in which we use our calibrated model (CM) to predict how PIEZO1 affects wound closure (left column) and wound edge length (right column) in simulated ControlGoF monolayers (gray), Piezo1-GoF monolayers without altered adhesion parameters (white), Piezo1-GoF monolayers with increased cell-cell adhesion (orange) and decreased cell-cell adhesion (blue). (B) Similar to A but using simulation results from DMSO-treated monolayers (gray), Yoda1-treated monolayers without altered adhesion parameters (white), Yoda1-treated monolayers with increased cell-cell adhesion (orange) and decreased cell-cell adhesion (blue). (C) Similar to C but using simulation results from ControlcKO monolayers (gray), Piezo1-cKO monolayers without altered adhesion parameters (white), and Piezo1-cKO monolayers with increased cell-cell adhesion (orange) and decreased cell-cell adhesion (blue). In A-C, n = 100 simulation results for each condition. To account for differences between control cases, data are normalized by rescaling to the mean of the corresponding control. Larger normalized wound closure indicates faster wound closure, while a smaller normalized wound closure indicates slower wound closure. Similarly, a larger normalized edge length indicates a more featured wound while a smaller normalized edge length indicates a flatter or less featured wound. Black check marks at the top of each plot condition indicate that simulation results match experimental trends while a red cross indicates the simulations fail to match the experiment results. Related to Table 3. For comparison with experimental data see Fig 1B, 1G and 1H. (TIFF) [file pcbi.1011855.s008.tiff]

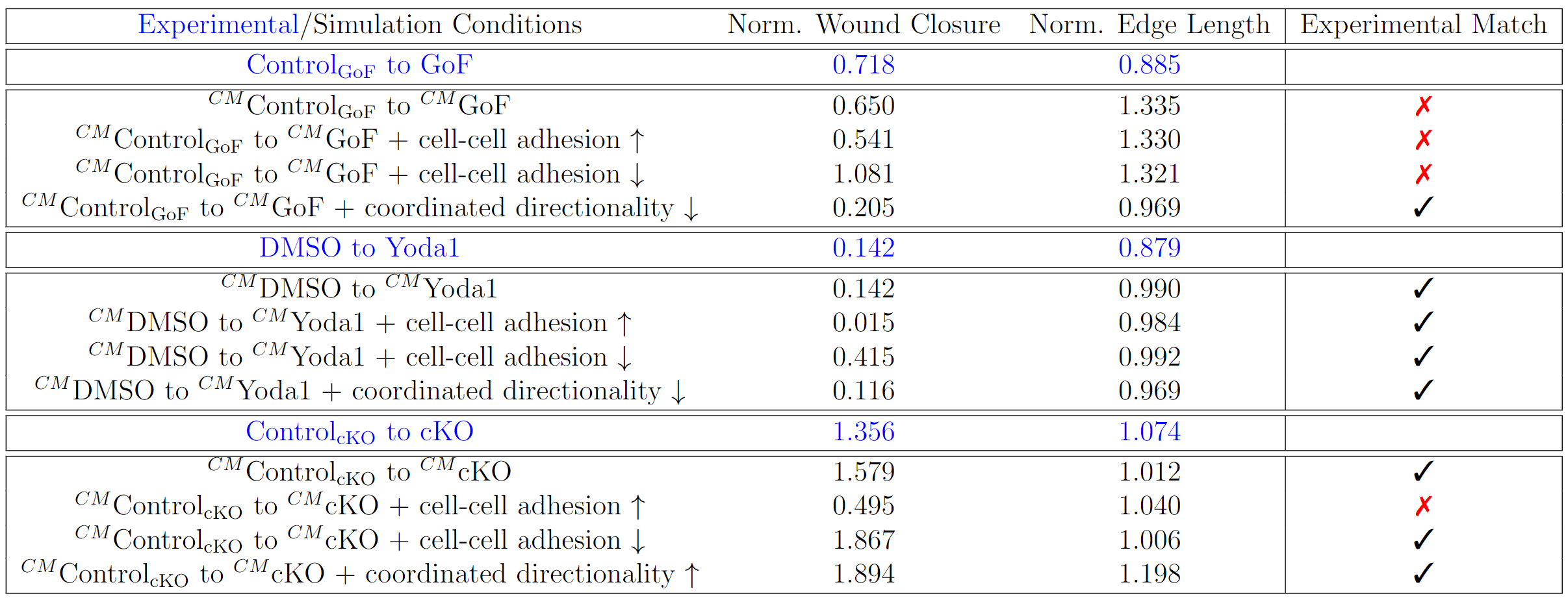

Supplement: S8 Fig — The table presents simulation results (in black, see Table 3 for qualitative results) obtained using the calibrated model (CM) to predict the impact of PIEZO1 on normalized wound closure and normalized edge length, altering adhesion and coordinated directionality parameters. The simulation results are quantitatively compared with the corresponding experimental results (in blue, see Table 1 for qualitative results). Model predictions are indicated in red font with a cross mark (✘) when they do not align with the experimental trends of increasing or decreasing values. Conversely, a check mark (✔) indicates that model predictions are consistent with the experimental trends. (TIFF) [file pcbi.1011855.s009.tiff]

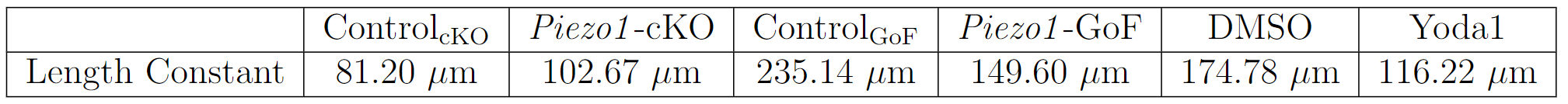

Supplement: S10 Fig — Summary table showing the length constant, or the distance at which the spatial autocorrelation value is estimated to reach 0.37, for each experimental condition. Length constants were calculated by fitting a curve to the respective experimental dataset. See also Fig 5. (TIFF) [file pcbi.1011855.s011.tiff]

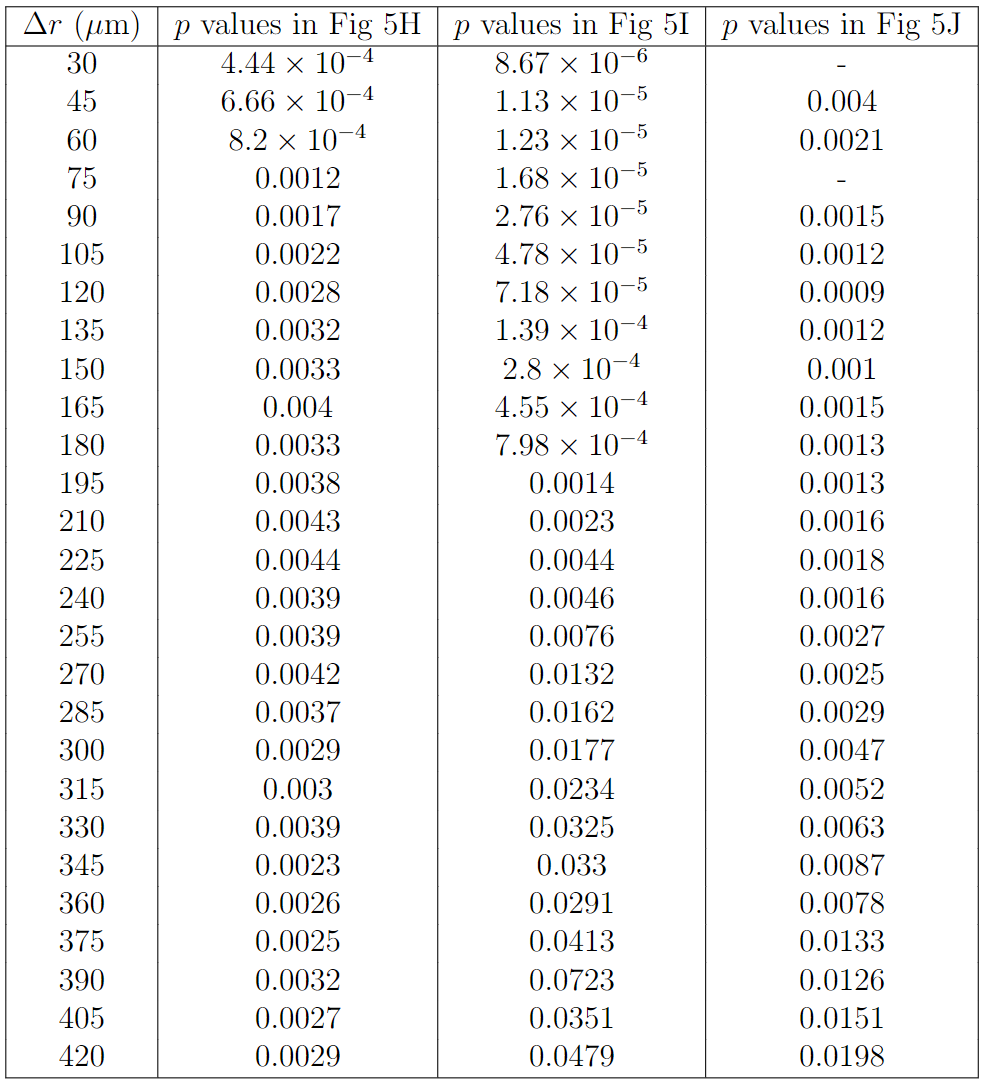

Supplement: S11 Fig — (TIFF) [file pcbi.1011855.s012.tiff]

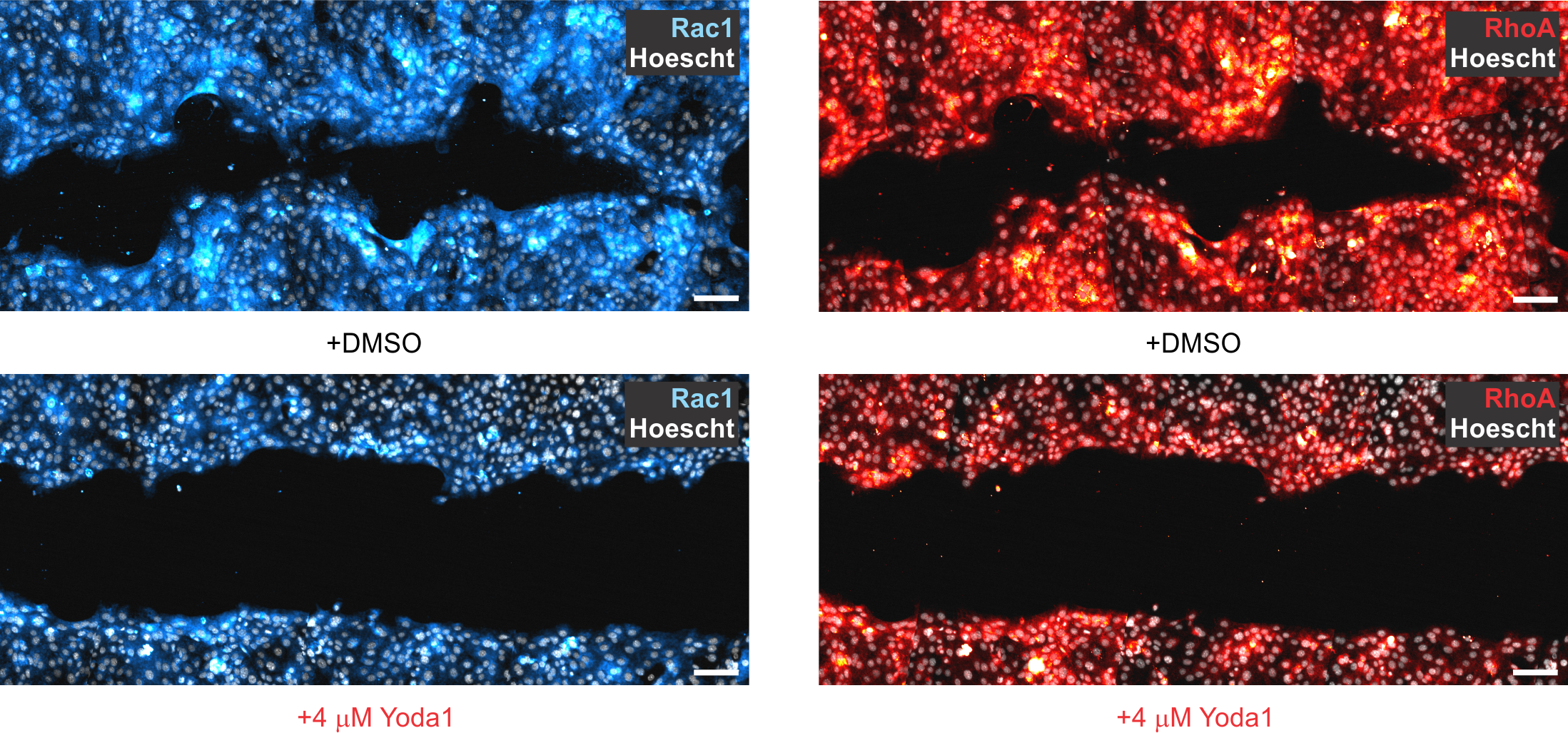

Supplement: S12 Fig — To explore a possible relationship between PIEZO1 and Rho GTPases we performed immunocytochemistry experiments for the Rho GTPases RhoA and Rac1 within healing monolayers. Scratch wounds were generated in keratinocyte monolayers and then immediately treated with either 4 μM Yoda1, or the equivalent amount of solvent DMSO. Keratinocyte monolayers were allowed to collectively migrate for 24 hours with the respective drug in the bath media before fixing and labeling monolayers. Shown above, representative images of healing keratinocyte monolayers immuno-labeled with antibodies against Rac1 (blue, left panels), and RhoA (red, right panels) 24 hours after wounding and treating monolayers with DMSO (top) and 4 μM Yoda1 (bottom). Increasing PIEZO1 activity through Yoda1-treatment decreases Rac1 and RhoA staining suggesting that PIEZO1 activity regulates Rho GTPase expression during keratinocyte collective migration. Scale bar = 100 μm. (TIFF) [file pcbi.1011855.s013.tiff]

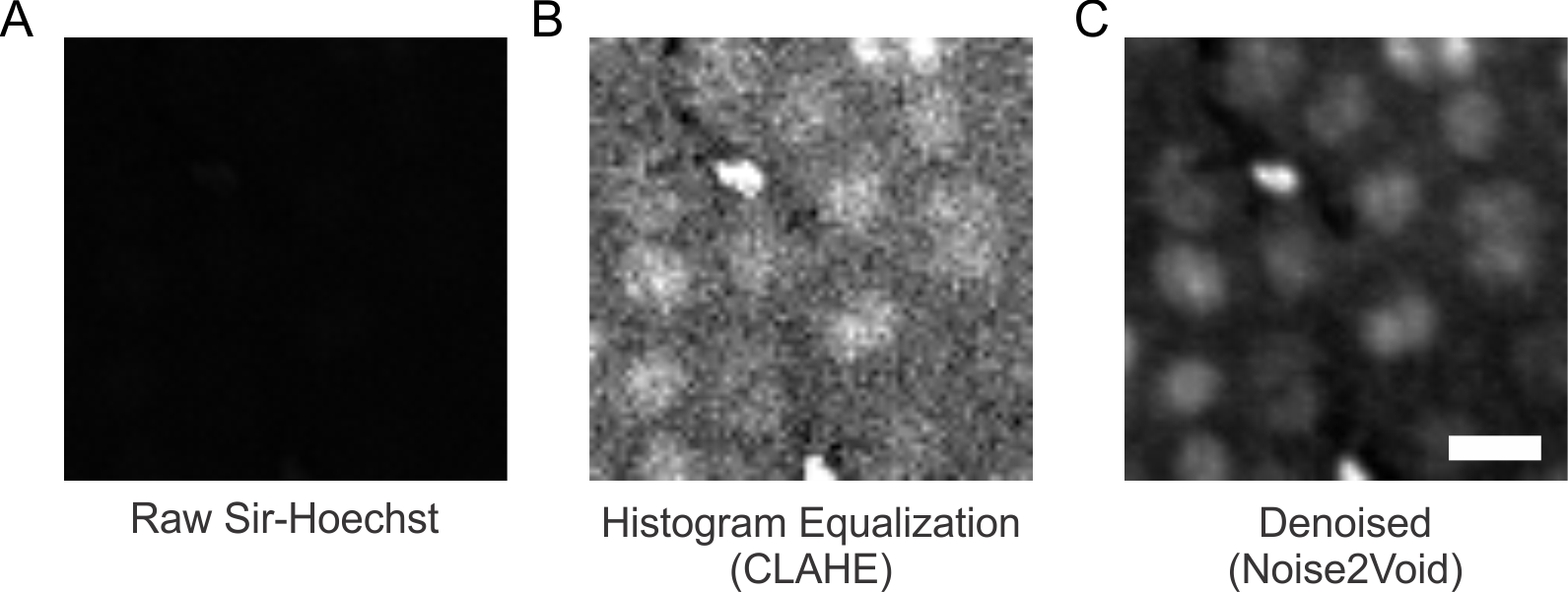

Supplement: S13 Fig — Representative images of processing steps to boost signal-to-noise ratio of (A) raw SiR-Hoechst images by first performing (B) histogram equalization using Contrast Limited Adaptive Histogram Equalization (CLAHE). (C) For some images, the denoising algorithm Noise2Void was used to further increase the signal-to-noise ratio of nuclei. Note: all images adjusted to the same brightness and contrast settings. Scale bar = 20 μm. (TIFF) [file pcbi.1011855.s014.tiff]

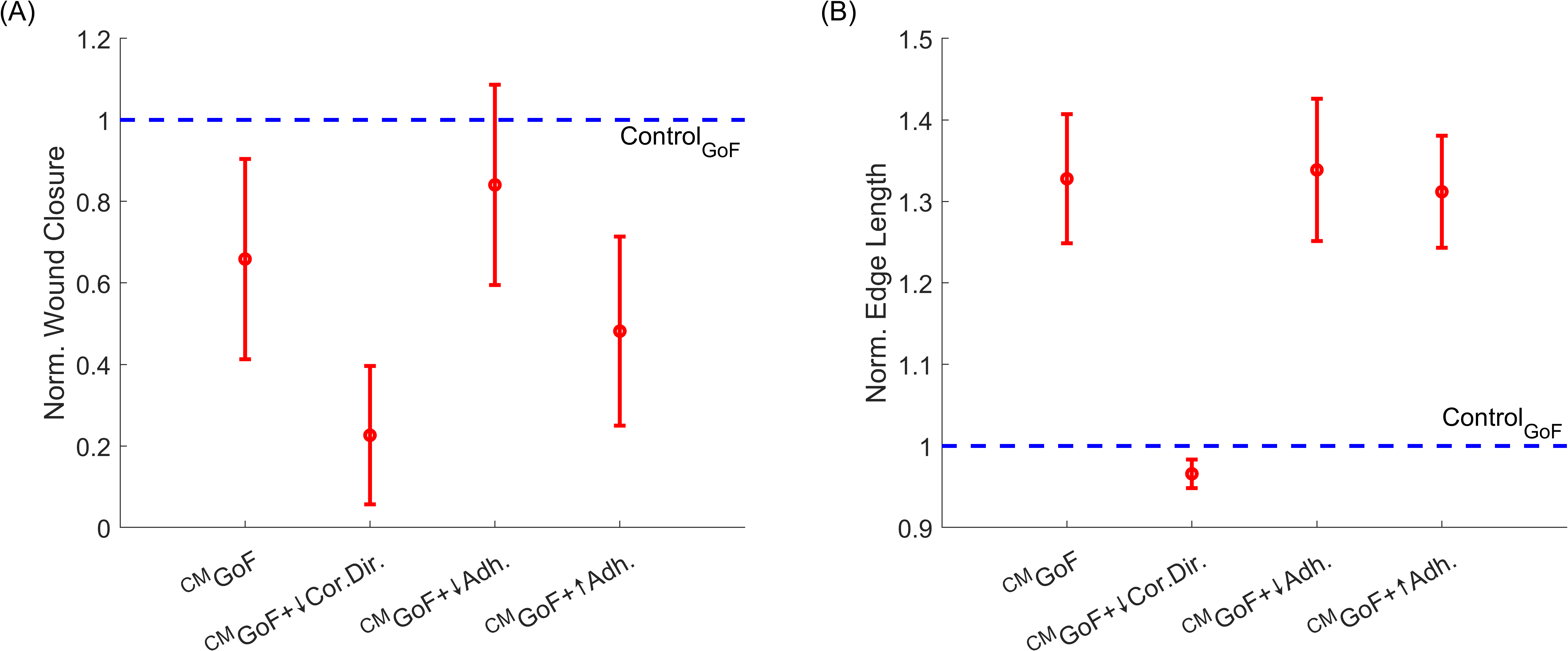

Supplement: S18 Fig — (A) Dot plots illustrate the mean of 2700 simulation results from the model using three values of the magnitudes of the retraction processes. They depict how PIEZO1 influences normalized wound closure in Piezo1-GoF monolayers compared to simulated ControlGoF monolayers (blue dashed line). The scenarios include Piezo1-GoF monolayers without altered coordinated directionality and cell-cell adhesion parameters (first column), Piezo1-GoF monolayers with decreased coordinated directionality (second column), Piezo1-GoF monolayers with decreased cell-cell adhesion (third column), and Piezo1-GoF monolayers with increased cell-cell adhesion (fourth column). See Section 8 in S1 Text for the details on the model parameters. Error bars indicate the standard deviation. (B) Similar to A but measuring the changes in normalized edge length instead of normalized wound closure. (TIFF) [file pcbi.1011855.s019.tiff]

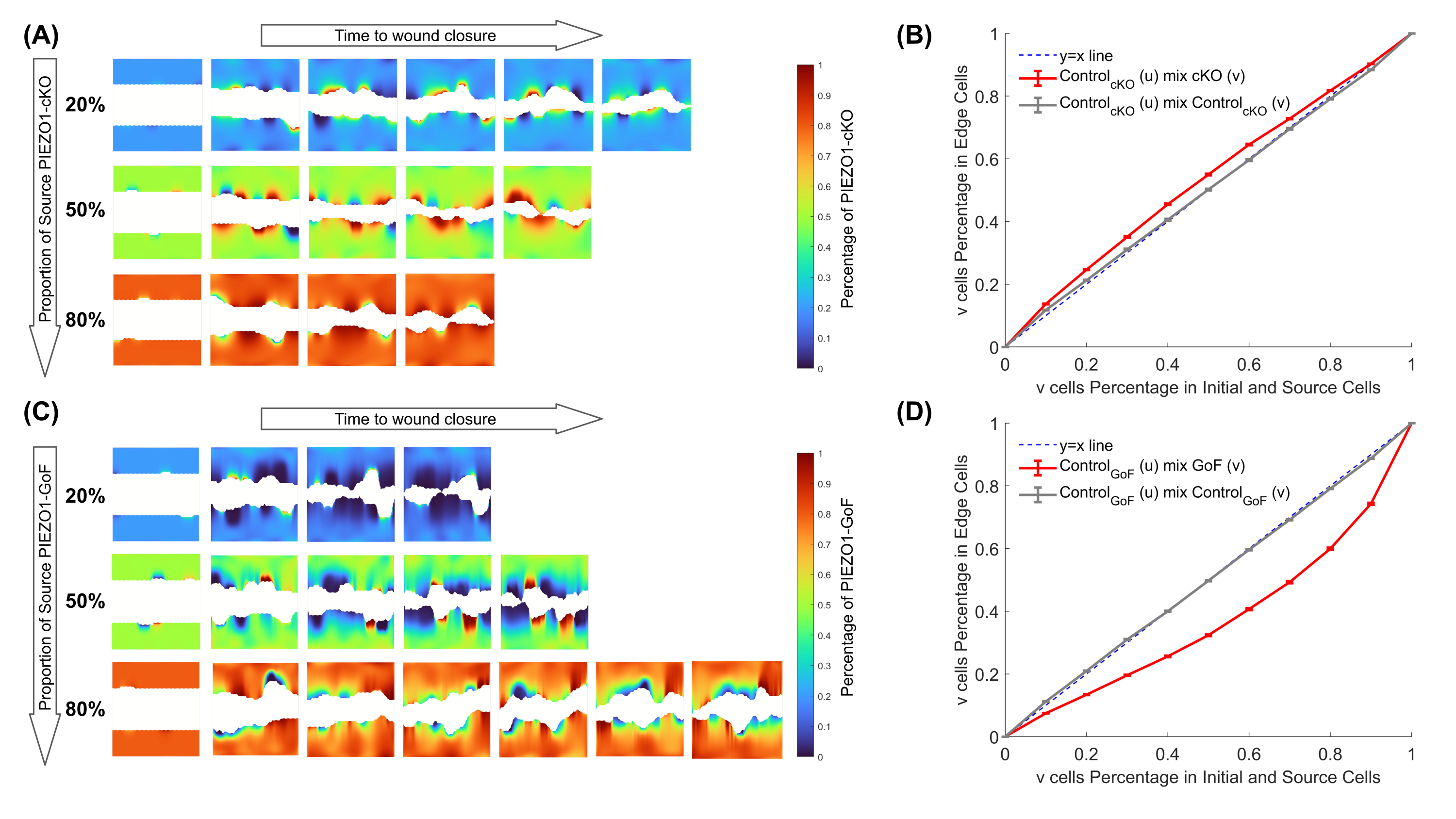

Supplement: S19 Fig — (A) and (B) show that cells with reduced PIEZO1 activity are overrepresented at the leading edge in mixed cell migration. (A) Snapshot of wound healing progression for a mixture of ControlcKO (u cells) and Piezo1-cKO (v cells) captured at equidistant time intervals, under varied initial and source cell conditions with Piezo1-cKO (v cells) percentages of 20% (top), 50% (middle), and 80% (bottom). Colored areas represent cell monolayers, with colors indicating the spatial distribution of Piezo1-cKO (v cells) percentage, while plain white areas denote cell-free space. (B) Line graphs illustrate the mean of 100 simulation results, displaying the percentage of Piezo1-cKO (v cells) cells in edge cells versus the percentage in initial and source cells. The red solid curve represents the mixing of Piezo1-cKO (v cells) with ControlcKO (u cells), the gray solid curve represents the scenario of mixing the same wild-type ControlcKO cells (i.e., u and v cells are both ControlcKO), and the blue dashed line signifies the y = x line. Error bars indicate the standard error of the mean. (C) and (D) Similar to (A) and (B), but involving mixtures of Piezo1-GoF (v cells) with their respective wild-type control (u cells). Piezo1-GoF (v cells) are underrepresented at the leading edge during migration. (TIFF) [file pcbi.1011855.s020.tiff]
